# Zirconium Carbide Produced by Spark Plasma Sintering and Hot Pressing: Densification Kinetics, Grain Growth, and Thermal Properties

**DOI:** 10.3390/ma9070577

**Published:** 2016-07-14

**Authors:** Xialu Wei, Christina Back, Oleg Izhvanov, Christopher D. Haines, Eugene A. Olevsky

**Affiliations:** 1 Department of Mechanical Engineering, San Diego State University, 5500 Campanile Dr., San Diego, CA 92182, USA; eolevsky@mail.sdsu.edu; 2 General Atomics, 3350 General Atomics Ct., San Diego, CA 92121, USA; tina.back@ga.com (C.B.); oleg.izhvanov@ga.com (O.I.); 3 US Army Armament Research Development Engineering Center, Picatinny Arsenal, NJ 07806, USA; christopher.d.haines2.civ@mail.mil; 4 Department of NanoEngineering, University of California, San Diego, 9500 Gilman Dr., La Jolla, CA 92037, USA; eolevsky@ucsd.edu

**Keywords:** zirconium carbide, spark plasma sintering, finite element simulation, grain growth, thermal properties

## Abstract

Spark plasma sintering (SPS) has been employed to consolidate a micron-sized zirconium carbide (ZrC) powder. ZrC pellets with a variety of relative densities are obtained under different processing parameters. The densification kinetics of ZrC powders subjected to conventional hot pressing and SPS are comparatively studied by applying similar heating and loading profiles. Due to the lack of electric current assistance, the conventional hot pressing appears to impose lower strain rate sensitivity and higher activation energy values than those which correspond to the SPS processing. A finite element simulation is used to analyze the temperature evolution within the volume of ZrC specimens subjected to SPS. The control mechanism for grain growth during the final SPS stage is studied via a recently modified model, in which the grain growth rate dependence on porosity is incorporated. The constant pressure specific heat and thermal conductivity of the SPS-processed ZrC are determined to be higher than those reported for the hot-pressed ZrC and the benefits of applying SPS are indicated accordingly.

## 1. Introduction

Spark plasma sintering (SPS), also known as field-assisted sintering or current-assisted sintering, is currently one of the most attractive rapid powder consolidation techniques. It has been evidenced that the Joule heating and the hydraulic loading acting in a SPS system allow the production of dense materials at lower temperatures and during shorter periods of time compared to SPS’ conventional counterpart technique—hot pressing [1,2,3,4]. Recently, SPS has been successfully utilized to consolidate ultra-high temperature ceramic (UHTC) powders, such as tantalum carbide [5], hafnium diboride [6], vanadium carbide [7], zirconium carbide [8], etc., into bulk articles with high densities and excellent properties. In addition to enhancing densification kinetics, the benefits from carrying out SPS of refractory powder-based materials include an impurities cleaning effect [9], early neck formation due to local overheating [10,11], and electric field-assisted grain size retention [12].

The aforementioned zirconium carbide (ZrC) is a typical UHTC possessing good high-temperature mechanical properties, excellent electrical and thermal conductivity, high melting point, and strong chemical resistance. It has been recently considered to be a promising candidate for high-temperature applications, such as furnace heating elements, plasma arc electrodes and nuclear cladding materials [13,14,15]. Although the implementations of these applications are still in progress, the attempts to consolidate ZrC powder started in the 1970s, when free-sintering and hot-pressing were employed for this purpose [16,17]. Due to ZrC’s high melting point (~3500 °C) and the inherent nature of the covalent Zr–C bonding, extremely high temperatures and long-term dwellings were usually required to obtain dense ZrC products via these techniques [18,19]. In spite of the inefficiencies, these conventional consolidation approaches have been often utilized in recent years [20,21].

Investigations on SPS of ZrC were initiated with retrieving high-density specimens under moderate conditions which had never been adopted previously in free-sintering or hot-pressing of ZrC. Sciti et al. reported that up to 98% relative density could be achieved at 2100 °C under 65 MPa within 3 min when conducting SPS of micron-grade ZrC powders [22]. Submicrometric zirconium oxy-carbide (ZrC_x_O_y_) powders were synthesized and consolidated by Gendre et al. at about 2000 °C [23], while the vacancies introduced by the carboreduction synthesis of such powders were considered to be the factors to facilitate densification [24]. Further enhancements of densification were implemented by employing post-processed nano ZrC powders in the SPS, in which the maximum processing temperatures could be way lower than 2000 °C [25,26]. These studies have suggested that the densification level achievable under SPS is significantly higher than the one obtained by carrying out conventional powder consolidation techniques.

ZrC powder densification mechanisms under SPS conditions were analyzed in the past. Gendre et al. used an empirical model to estimate the stress exponent and the activation energy in SPS of synthesized ZrC_x_O_y_ powder under different loads [23]. This model has been modified recently by Antou et al. with separating intermediate and final sintering stages when investigating the mechanisms contributing to the densification [27]. Wei et al. determined the densification mechanisms of commercial ZrC powder under SPS conditions, in which a densification equation based on the continuum theory of sintering has been used [28]. By carrying out a regression of the obtained equation to the experimental densification data, the strain rate sensitivity and activation energy of the employed ZrC powder were properly assessed [8]. All studies indicated that ZrC exhibits high activation energy and power law creep behavior during the SPS process.

Microstructure coarsening during the final stage of sintering was also observed by Gendre et al., in which the authors attributed this phenomenon to the onset of the pore-grain boundary separation [23]. However, the grain growth mechanism has not been unambiguously identified in that study. Temperature and electric current distributions during SPS of ZrC specimens were also analyzed by a finite element simulation [29]. Despite the fact that porosity of the studied ZrC specimen and the electric contact resistance had not been taken into consideration, a large temperature gradient was identified between the specimen and the SPS tooling area (to which the temperature measuring pyrometer has been focused). This thermal non-uniformity, as stated by the authors, was due to the non-uniform current density distribution in the SPS tooling system as well as the radiative heat loss at the outer surfaces of SPS tooling. It is, therefore, necessary to characterize these thermal effects before analyzing mass transfer and deformation mechanisms in SPS of powder materials.

Both partially and fully dense ZrC products can be utilized for various applications but the respective product service conditions are usually associated with high temperatures. Thermal properties, such as constant pressure specific heat capacity and thermal conductivity of ZrC are, therefore, critical to its potential applications. Measurements conducted a few decades ago on hot-pressed ZrC samples indicated that both heat capacity and thermal conductivity of ZrC increase with temperature [30,31]. However, thermal properties of the SPS processed ZrC have not been reported so far. In addition, the uses of high temperature ceramics sometimes require keeping certain levels of residual porosity in the products (for example, to accommodate volume swelling). In these cases, the specimen’s thermal properties largely depend on its relative density because the volume fraction of voids directly determines the amount of substance involved in heat transfer.

In this study, commercial ZrC powders have been subjected to SPS treatments under various processing conditions to produce specimens with a wide range of densities. Conventional hot pressing has also been utilized to consolidate ZrC powder, in which the obtained densification kinetics and microstructures are compared to these retrieved from SPS of ZrC under similar heating and loading profiles. The specimen’s temperature is determined using finite element method by correlating the simulated temperature inside the powder specimen with respect to the pyrometer measured temperature at the die surface. The resulting specimen’s temperature is utilized to investigate the grain growth mechanism during the final stage of SPS. Both densification and grain growth are studied by hiring recently-developed models [8,32]. The constant pressure specific heat capacity and thermal conductivity of the SPS-processed specimens are measured with respect to temperature, up to 1100 °C. The obtained thermal properties are compared to the reported ones, taking into consideration the relative density level.

## 2. Materials and Experiment

### 2.1. Starting Powders

**Figure 1:**
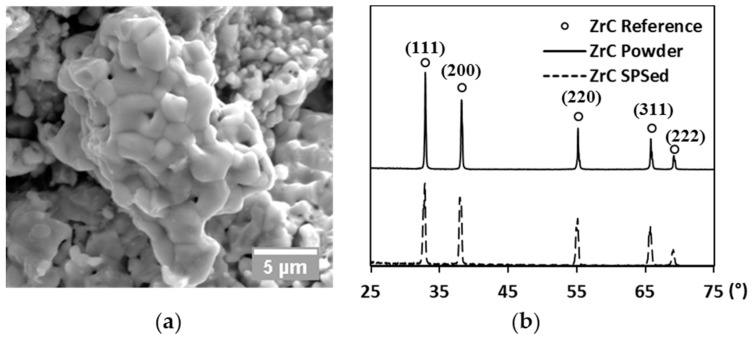
(**a**) SEM image of the raw powder; and (**b**) XRD patterns of raw powder (solid line), the SPS-processed specimen (dashed line), and reference peaks (ring markers), respectively.

A commercial zirconium (IV) carbide powder (99% metal basis, Sigma-Aldrich Co., St. Louis, MO, USA) was chosen as the tested material in the present study. The as-received powder was first subjected to ultra-sonication (2510 ultra-sonic cleaner, Branson Corp., Danbury, CT, USA) for de-agglomeration. The raw powder was then analyzed by scanning electron microscopy (SEM, Quanta 450, FEI Co., Hillsboro, OR, USA) to examine its morphology. As shown in Figure 1a, a single particle exhibits a polycrystalline structure with inter- and intra-granular pores present. The average grain size of the raw powder is around 1 µm. X-ray diffraction (XRD, X’Pert Pro, PANalytical B.V., Almelo, The Netherlands) of the raw powder was performed using copper as target, diffracted patterns (solid line) are compared to reference peaks (ring markers) along each diffracted plane in Figure 1b. Additionally, the lattice parameter of the starting powder was estimated at every diffracted plane to give an average value of 4.698 Å, which only showed a negligible difference in comparison to the theoretical value (4.699 Å, [31]). The XRD analysis, therefore, has identified the raw powder was very close to the stoichiometry of ZrC.

### 2.2. Consolidation of Zirconium Carbide Powder

All SPS experiments were performed using a Dr. Sinter SPSS-515 furnace (Fuji Electronic Industrial Co. Ltd., Kawasaki, Japan) with a pulse duration of 3.3 ms and on/off pulse interval of 12:2. For each SPS experiment, 4 g of ZrC powder were used. A 15.3 mm graphite die and two 15 mm graphite punches (I-85 graphite, Electrodes Inc., Santa Fe Springs, CA, USA) had been aligned by inserting well-cut 0.15 mm graphite paper (Fuji Electronic Industrial Co., Ltd., Kawasaki, Japan) in between. The weighted powder was then carefully loaded into the graphite tooling and pre-compacted at room temperature under 3 kN. The geometrical dimensions of a specimen at this point were then used to calculate its green density.

SPS runs were conducted with the maximum processing temperature ranging from 1600 °C to 1800 °C. The following heating profile was used: (i) 6 min from room temperature to 580 °C, 1 min from 580 °C to 600 °C and holding at 600 °C for another 1 minute; (ii) 100 °C/min to 1600 °C and 50 °C/min to target temperature; (iii) dwelling at peak temperature; and (iv) cooling down to 1000 °C and powering off the machine. The temperature was monitored by a digital pyrometer pointing at the lateral surface of the die. The hydraulic uniaxial pressure was consistently applied from the beginning to the end of the consolidation process. The real-time processing parameters, such as temperature, applied load, and axial displacement, were automatically logged by the SPS device.

Hot pressing of the same ZrC powder was carried out using a 50 t hot press furnace (Oxy-Gon Industries, Epsom, NH, USA). The uniaxial pressure was set to 55 MPa. The heating rate was 13 °C/min to 1900 °C. Isothermal holding at 1900 °C was 60 min. In order to make a comparison, “control” SPS runs with same external pressure, heating rate, and holding time were also implemented. By considering the existence of the temperature gap between the specimen and the outer die surface during SPS [33], the peak processing temperature in “control” SPS runs was adjusted to 1600 °C. Such an adjustment aimed at making the actual temperature which the specimen experienced during SPS to be comparable to the one that used in hot pressing (see also Section 3.2). Therefore, the hot pressing and the SPS of ZrC were able to be conducted with imposing similar heating and loading profiles to the powder specimens.

An argon atmosphere was utilized in all SPS and hot-pressing experiments in order to prevent the furnace chamber and the heating elements from being overheated. Graphite tooling was wrapped by carbon felt to reduce heat loss through thermal radiation in SPS runs. For every selected processing profile, an additional run was conducted in the absence of powder. The obtained axial displacement data from this idle run was subtracted from the one retrieved from the real run to provide the true axial shrinkage of a specimen. Every individual experiment was repeated at least twice to ensure the reproducibility of the results.

### 2.3. Characterization of Processed Specimens

The spark plasma-sintered ZrC specimens have been characterized to reveal their density, open porosity, phase composition, and grain size. All obtained specimens were ground with abrasive SiC paper to remove the adherent graphite foil from their outer surfaces. A specimen’s density was first calculated using a geometrical method. If the ratio of the geometrical density of a specimen to the theoretical density of ZrC (6.7 g/cm^3^), i.e., the relative density, was more than 90%, the Archimedes method was also applied to reconfirm the obtained value of the relative density. The true axial shrinkage was employed to evaluate the densification kinetics of a specimen with respect to the processing time by assigning a constant radius to the specimen during SPS processing. Open porosity was determined using a helium pycnometer (AccuPyc 1330, Micromeritics Corp., Norcross, GA, USA) by taking into account the difference between apparent and pycnometric relative densities.

After density and open porosity measurements, specimens SPSed at 1700 °C were evenly cut by a precision saw (IsoMet 1000, Buehler, Lake Bluff, IL, USA). The two halves of a specimen were hot-mounted in Bakelite powder with cross-sectional surfaces facing out and subsequently polished with the assistance of a colloidal diamond suspension. Well-polished samples were first analyzed by XRD (X’Pert Pro, PANalytical B.V., Almelo, The Netherlands) to retrieve specimens’ phase compositions after SPS consolidation. Then, the polished surfaces were etched for 2 min using HF:HNO_3_:H_2_O solution in a volumetric ratio of 1:1:3 in order to have a better reflection of their grain geometries in microstructural characterizations. The obtained micrographs were analyzed by an image software (ImageJ 1.5 g, NIH Image, Bethesda, MD, USA) to calculate the specimen’s average grain size based on the mean linear intercept method with a correction factor of 1.5 [34].

### 2.4. Temperature Evolution in SPS of ZrC

The finite element simulation using COMSOL^®^ Multiphysics software (Comsol Inc., Burlington, MA, USA) was employed to couple electric current and consequent Joule heating phenomena in the implementation of thermal aspects of the employed SPS system. The coupled equations are:

(1) $$\rho_{eff}C_{p}\frac{\partial T}{\partial t}-\nabla \cdot \left(k_{T}\nabla T\right)=h$$

where $\rho_{eff}$ is the density $\left(kg/m^{3}\right)$; $C_{p}$ is the heat capacity (*J/kg/K*) and $k_{T}$ is the thermal conductivity (*W/m/K*). h denotes the heat generated by the flowing electric current:

(2) $$h=\left|J\right|\left|E\right|=\lambda {\left|\nabla V\right|}^{2}$$

where J is the electric current density $\left(A/m^{2}\right)$ and E is the intensity of the electric field (V/m); Parameters λ and ∇V correspond to the electric conductivity $\left(\Omega^{-1}\cdot m^{-1}\right)$ and the gradient of electric potential (V/m), respectively. The electric contact resistance between the graphite tooling components was included as:

(3) $$\vec{n}\cdot {\vec{J}}_{ec}=\frac{1}{R_{ec}}\left(V_{1}-V_{2}\right)$$

where $\vec{n}$ is the normal to the contact surface; ${\vec{J}}_{c}$ is the generated current density at the contacts $\left(A/m^{2}\right)$; $R_{ec}$ is the electric contact resistance $\left(\Omega \cdot m^{2}\right)$, which has been experimentally derived with respect to the same tooling system [35]; $V_{1}$ and $V_{2}$ are the electric potential at any two contact surfaces. The effects of thermal contact resistance was implemented by applying the equations developed in [35,36]. The role of horizontal thermal contact resistance was ignored in the simulation as it has been previously determined that its effects on the temperature field are negligible if high pressure is applied [37].

Thermal and electric properties, including the temperature dependence, of the utilized graphite tooling, followed the expressions previously used by Olevsky et al. [32]. ZrC specimen’s thermal and electric properties during processing are given in Table 1 as functions of porosity, *θ*, and temperature, *T* (*K*). ZrC’s thermal properties were selected in accordance with [15,31].

**Figure 2:**
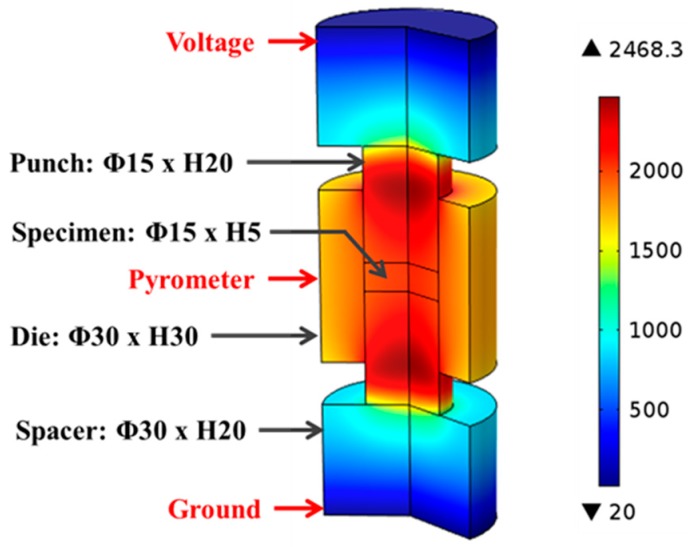
Geometrical model and temperature distribution in finite element simulation, Unit: °C.

The SPS machine’s logged voltage readings were converted to their root mean square values and interpolated with respect to processing time to provide continuous inputs for the entire modeling process. Figure 2 illustrates the major portion of the tooling-specimen system, which was built as an axial-symmetric model in COMSOL^®^ with specifying the dimensions of each component. During the simulation, the electric potential was introduced at the top electrode (not included in Figure 2), while the bottom one was grounded.

The simulated temperature of the control point at which the temperature measuring pyrometer has been focused was compared to the one obtained from the experiment. These two sets of data have to be in good agreement with each other in order to confirm the reliability of the modeling results and retrieve the specimen temperatures from the simulation. The radial temperature gradient was then calibrated by correlating the calculated control point specimen temperatures with the pyrometer temperatures measured at the control point to allow a suitable comparison of densification kinetics between SPS and hot pressing.

### 2.5. Measurement of Thermal Properties

A series of SPS processed specimens with relative densities ranged from 73.9%–93.3% were further ground to 6 mm diameter by 1 mm thickness disks for thermal property tests. The heat capacity measurements were conducted under constant pressure using the differential scanning calorimeter (DSC 404 F1 Pegasus, Netzsch Co., Selb, Germany) along with the corresponding laser flash apparatus (LFA 427, Netzsch Co., Selb, Germany). The thermal diffusivity was determined by measuring the temperature change on the upper surface of the sample caused by a pulsed laser flash acting on its lower surface. Then, the thermal conductivity was considered to be the product of the sample’s heat capacity, density, and its thermal diffusivity calculated by the laser flash apparatus [38]. All tests were performed at every 100 °C interval from room temperature to 1100 °C in an argon atmosphere.

## 3. Discussion

### 3.1. Densification Kinetics

**Figure 3:**
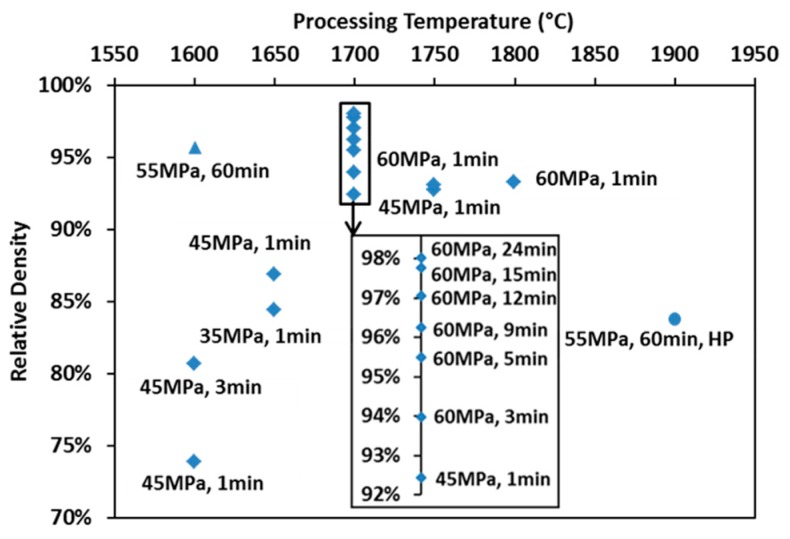
Map of relative densities for specimens prepared under various processing conditions.

The final relative densities of the spark plasma sintered specimens are mapped with processing parameters in Figure 3 (diamond markers). Relative densities of specimens prepared at 1700 °C have been rescaled to be more visible. The density of the hot-pressed specimen (round marker) is also present in comparison to that of the spark plasma sintered one subjected to similar heating and loading profiles (triangle marker). An enhancement in any of the processing parameters leads to an increase of the product’s final density. The X-ray diffracted pattern of the SPS-processed specimen is compared to that of the raw powder and to the reference peaks in Figure 1b. The lattice parameter of the SPS specimens was calculated to be ~0.2% larger than that of the raw powder. Such an augmentation might be caused by the free carbon in the raw powder reacting with ZrC during SPS. Since the amount of lattice parameter change could only influence the stoichiometry and lower the theoretical density negligibly, it was considered as minor in the calculation of relative density.

**Figure 4:**
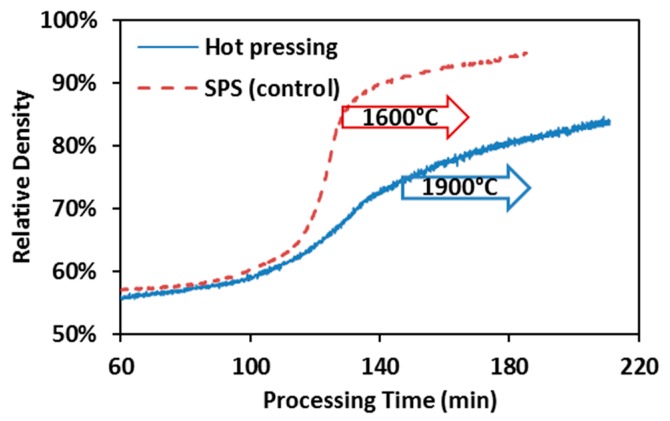
Hot pressing vs. control SPS of ZrC: Comparison of densification kinetics, under 55 MPa and 60 min holding.

Densification kinetics of spark plasma sintering and hot pressing of ZrC is summarized in Figure 4 with the arrows indicating the onset of the isothermal dwelling. The densification curve of the control SPS appears to possess less data points than that of hot pressing, which is due to the fact that the peak processing temperature in control SPS runs (1600 °C) was intentionally selected to be lower than that in hot pressing (1900 °C). As a result, the hot pressing spent more time to achieve the target temperature. In the control SPS runs, fast densification has already started before the maximum processing temperature has arrived. While in the hot pressing, it is hard to identify the fast densification period until the end of the entire process. Therefore, hot pressing has been evidenced to be much less efficient than SPS in processing ZrC powders.

**Figure 5:**
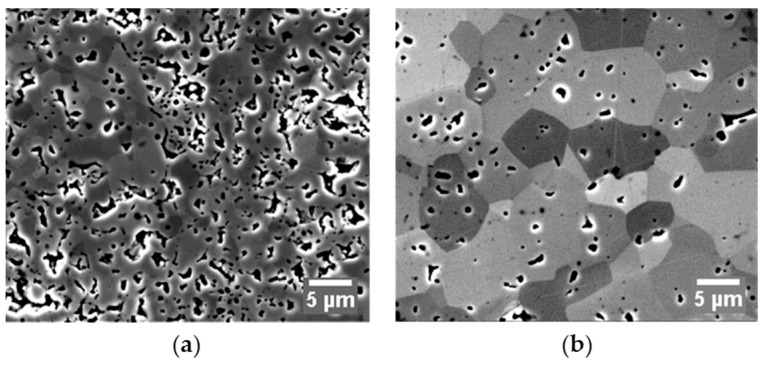
Microstructures of ZrC processed by (**a**) hot pressing at 1900 °C; and (**b**) control SPS at 1600 °C under 55 MPa and 60 min holding. The contrast between grains indicates the grain orientations.

Specimens prepared by hot pressing and control SPS processes also gave quite different microstructures when being observed under SEM. As shown in Figure 5, the hot-pressed specimens (Figure 5a) possess a porous structure with visible inter-particle contacts and insignificant signs of grain coarsening. However, under the same magnification, a much more consolidated morphology is present in the SPS-processed specimen (Figure 5b) with clear exhibitions of large grains, while only isolated individual pores are displayed in the matrix.

### 3.2. Temperature Evolution in SPS of ZrC

**Figure 6:**
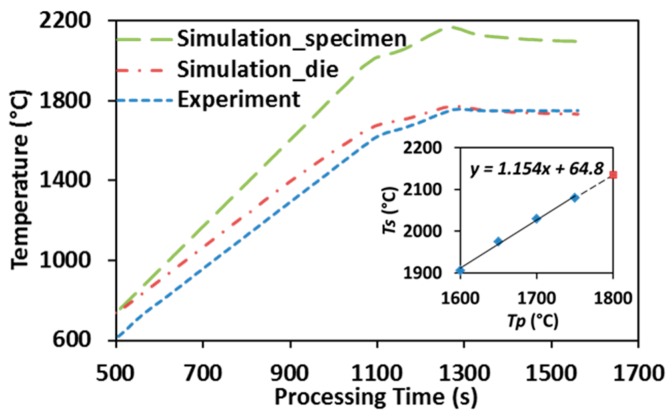
Simulation vs. experiment: temperature evolution in SPS of ZrC (up to 1750 °C).

Figure 2 illustrates the temperature distribution obtained from conducting finite element simulation of SPS of ZrC at 1750 °C with color bar indicating the temperature levels on the right. One can see that the temperature is non-uniformly distributed in the entire system. Simulated temperature values at the point of the pyrometer measurement (long-dash line) and the average temperature in the volume of the specimen (dot-dash line) are plotted in Figure 6, including experimentally-obtained temperature data as a reference (dashed line). The evolution of simulated temperatures at the pyrometer spot show a good agreement with that of the experimental readings, acceptable discrepancies at low temperature range were most likely caused by the lagging of the utilized SPS machine, as well as the radiative heat loss during the rapid heating (100 °C/min) period. However, the specimen’s temperatures extracted from the simulation are significantly higher than those retrieved from the experiment and the gaps between these two sets of data keep growing as processing temperature rises. This non-uniform temperature distribution in the tooling system is a common phenomenon in the SPS process and should be carefully assessed [32,39].

After plotting the simulated specimen’s temperatures (*T_s_*) with respect to the pyrometer-measured processing temperatures (*T_p_*), as shown in the embedded graph of Figure 6, a nearly linear relationship was obtained with processing temperature varying from 1600 °C to 1750 °C. The trend line is similar to the one that has been attained by Antou et al. [29] via finite element simulation, as well as Kelly and Graeve through conducting SPS runs with both top and side pyrometers attached [40]. Additionally, the extrapolation of the obtained relationship has been demonstrated to be able to predict the specimen’s temperature when higher SPS temperature is imposed (dashed extension line). Therefore, the temperature experienced by a ZrC specimen subjected to different SPS processing temperatures can be estimated and subsequently used in characterizing densification mechanisms (Section 3.3) and grain growth (Section 3.4).

### 3.3. Densification Mechanisms in SPS and Hot Pressing of ZrC

In regard to the sintering stages, the hot-pressed ZrC ended up with an 84% relative density which corresponded to the intermediate sintering stage, while the control SPS ZrC has evolved into the final sintering stage with 95% relative density being achieved. Densification mechanisms incorporated in control SPS and hot pressing of ZrC powders under similar heating and loading profiles were investigated to explain the observed different densification kinetics. An analytical/numerical approach for determining the creep coefficients of powder based materials subjected to hot consolidation in a rigid die has been developed recently, in which an analytical densification equation was derived based on the constitutive equation of sintering, as [8]:

(4) $$\dot{\theta }=\frac{d\theta }{dt}=-\frac{A_{0}}{T}exp\left(-\frac{Q}{RT}\right){\left(\sigma_{z}\right)}^{\frac{1}{m}}{\left(\frac{3\theta }{2}\right)}^{\frac{m+1}{2m}}{\left(1-\theta \right)}^{\frac{m-3}{2m}}$$

where $\sigma_{z}$ is the applied axial pressure (*Pa*); *T* is specimen’s absolute temperature (*K*); *m* is the strain rate sensitivity; *Q* is the activation energy (*J*/*mol*); and $A_{0}$ is a combined material constant. The creep coefficients, *m*, *Q*, and $A_{0},$ can be determined through numerically solving Equation (4) in regression to the experimental densification data. A detailed elucidation of such an analysis has been given in [8].

For hot pressing of ZrC, the densification data from the entire isothermal holding stage was selected as the benchmark in regression analysis with the relative density ranging from 75%–84%. At the same time, the selection of densification data from the control SPS runs was taking both the ramping-up and the holding periods into account with relative density increasing from 75%–95%. These selections ensured the same starting porosity (~25%) in both cases. It should be noted that, according to the selected range of relative density, the hot pressing only corresponds to the intermediate sintering stage, while the control SPS includes two sintering stages with the intermediate one preceding the final one [41], and the densification rates associated with these two stages are different (see also Figure 4). Therefore, the study of densification mechanism involved in the intermediate stage was individuated from the one that engaged in the final stage. This approach enabled comparing densification mechanisms incorporated in hot pressing and SPS during the same sintering stage and extended the investigating approach that employed by [8], in which the intermediate and final SPS stages were counted together.

**Figure 7:**
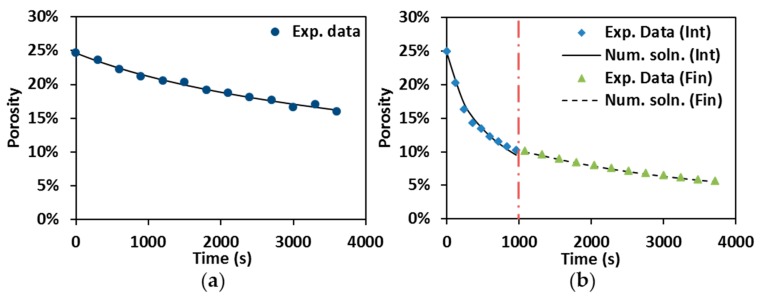
Numerical solution vs. experimental data: (**a**) hot pressing at 1900 °C; and (**b**) control SPS at 1600 °C, under 55 MPa and 60 min holding.

Numerical solutions (Num. soln) are compared to experimental data (Exp. data) in Figure 7. The numerical results are in good agreement with the representative experimental results as shown in Figure 7a, which reveals the reliability of Equation (4) for describing porosity evolution in hot pressing. Porosity evolution during the control SPS has been first split into intermediate (Int) and final stages (Fin) in order to individuate the densification behavior, and then these two stages were put together in one plot (Figure 7b). The discontinuity of the numerical solution at the junction point between the two stages (vertical dot-dash line) reflects the change of creep coefficients.

Optimal creep coefficients used in regression analysis are summarized in Table 2 based on the corrected specimen temperature (see Section 3.2). All of the values of the strain rate sensitivity, *m*, no matter which consolidation technique was used, fall into the range from 0.33 to 0.5. The densification involved in SPS and hot pressing of ZrC is most likely to be grain boundary sliding (m=0.5) associated with dislocation glide (m=0.3) controlled creep [42,43]. Although the *m* value obtained for the hot pressing was slightly smaller than that of the control SPS obtained for the same sintering stage, the control SPS rendered a significantly lower *Q* value than the one that the hot pressing provided. Comparatively higher strain rate sensitivity and lower activation energy retrieved from the SPS runs can be attributed to the contribution of electric current, improving the neck growth between particles. Although quantitative evaluations of the current effect in the SPS process are still ongoing [1,2,3], as shown in [10,11], the inter-particle necks have been observed to be formed at the early SPS stages. Extra atomic diffusional paths created in this manner substantially accelerated the activation of the plastic flow. At the same time, during hot pressing, the inter-particle necks (see Figure 5) appeared to start growing during the intermediate stage and, thus, provided less support for mass transport; therefore, higher energy was required in the case of hot pressing. The creep coefficients of the control SPS at the intermediate and final sintering stages are nearly identical, except for slightly different values of the activation energies. This difference might be related to the underestimation of the specimen’s temperature and the viscous analogue of the shear modulus due to the influences of porosity during the final stage of SPS.

### 3.4. Grain Growth and Microstructures of SPS Processed Specimens

**Figure 8:**
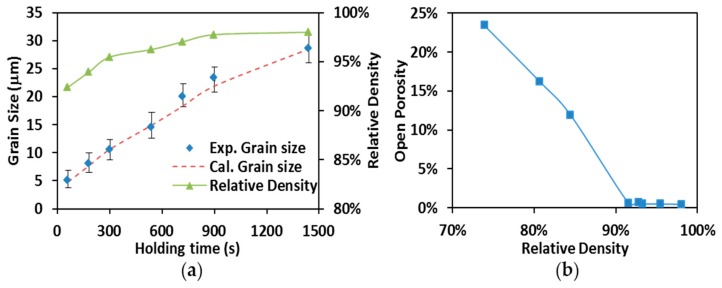
(**a**) Grain size vs. relative density (SPS at 1700 °C); (**b**) open porosity vs. relative density.

Average grain sizes (diamond markers) and relative densities (solid line with triangle markers) obtained from specimens produced by SPS processing at 1700 °C are present in Figure 8a with holding time up to 1440 s (24 min). One can see that the increase of the relative densities is accompanied by the augmentation of the grain sizes. Nevertheless, the grain growth appears to be more significant compared to the density evolution. As shown in Figure 8a, the specimens’ relative densities range (from 92.3% to 98.1%) indicates the sintering of ZrC has evolved into the final stage when isothermal dwelling started at 1700 °C. During this stage, when the saturation of the temperature level on densification is shown, the processing temperatures still substantially facilitated the grain growth as holding time proceeds [44].

Chaim stated that, besides temperature and time, the grain growth in SPS of porous ceramics is also controlled by the pore mobility [45]. An equation that includes the dependence of the grain growth on these factors was proposed by Olevsky et al. as [32]:

(5) $$G^{p}=G_{0}^{p}+k_{0}t{\left(\frac{\theta_{c}}{\theta +\theta_{c}}\right)}^{\frac{3}{2}}exp\left(-\frac{Q_{G}}{RT}\right)$$

where $G_{0}$ is the initial grain size; p is the grain growth exponent; $k_{0}$ is the grain growth constant; $\theta_{c}$ is the critical porosity which reflects the transition from open to close porosity and $Q_{G}$ is the activation energy for grain growth (*J*/*mol*).

By using the simulation approach provided in Section 3.2, the specimen’s temperature, *T*, was evaluated to be 2303 K (~2030 °C) which is corresponding to a pycnometer-measured temperature of 1700 °C. The critical porosity, $\theta_{c}$, was determined through the open porosity measurements. The specimen’s open porosities are plotted with respect to their relative densities in Figure 8b. The decrease of open porosity suddenly turns into a plateau with open porosity close to zero after relative density reaches 93%, indicating the open pores in these specimens are nearly gone. The turning point Figure 8b was, therefore, considered to be the moment of transition from open porosity to close porosity and the value of $\theta_{c}$ was set to 0.07 in the evaluation of other grain growth coefficients.

An Excel^®^ Solver program (Microsoft, Redmond, WA, USA) was used to assess the values of p, $k_{0}$, and $Q_{G}.\;$By iteratively optimizing these values, as demonstrated by the dashed line in Figure 8a, Equation (5) produced a set of calculated grain sizes which consistently agree with the ones obtained from the experiments. Additionally, the coefficient optimization gave a grain growth exponent of p≈2, which corresponds to the grain boundary diffusion controlled grain growth [45]. The observed insignificant change in density during the final holding stage in the present study is in agreement with the study of Djohari et al., in which the grain boundary diffusion has been described as a cause of virtually little densification in the later stage of sintering [46]. Furthermore, the activation energy for grain growth was estimated to be 290 kJ/mol. This value is way lower than the activation energies found for zirconium lattice diffusion (720 kJ/mol, [47]), for carbon bulk self-diffusion (470 kJ/mol, [48]) in ZrC_x_ and for creep-introduced densification (576 kJ/mol, see also Table 2), suggesting that the grain growth was preferred during the final stage of SPS of ZrC compared to other mechanisms.

**Figure 9:**
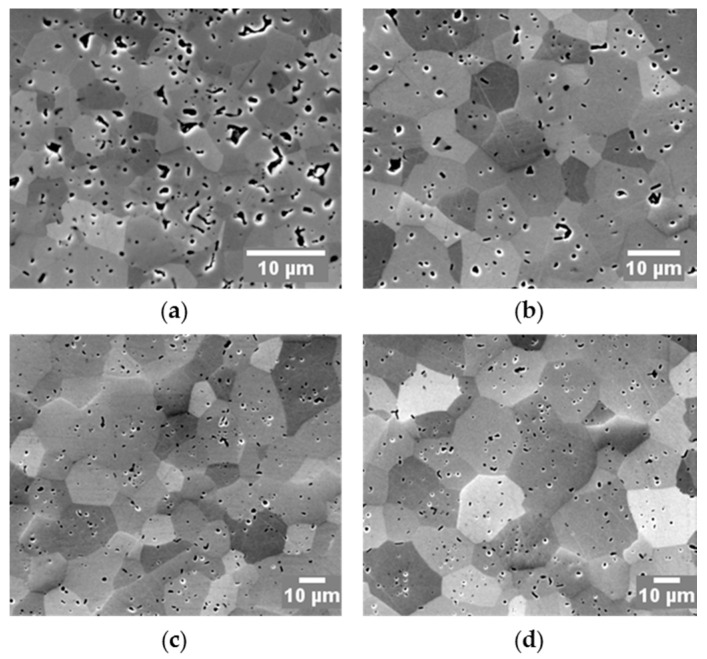
Microstructures of SPS-processed specimens at 1700 °C with: (**a**) 1 min; (**b**) 9 min; (**c**) 15 min; and (**d**) 24 min holding time, all under a pressure of 60 MPa.

The representative micrographs of specimens’ cross-sectional surfaces are illustrated in Figure 9, from where a direct impression of how grains interact with inter-granular pores at the triple junctions can be obtained: the grain growth gradually contributes to the process of pore closure. It appears that the densification can benefit from the grain growth to a certain degree in the final stage. However, this phenomenological observation could be complemented by nano- or atomic- scale analyses to reveal the actual mass transfer mechanism (motions of grains or dislocations). The existence of the amount of intra-granular pores in the microstructures of all specimens is possibly due to: (i) internal pores from initial powder (see also Figure 1a); (ii) high-temperature pore formation mechanisms proposed by Kelly and Graeve [40]. The contrast between grains indicates the grain orientations. The contrast difference seems to become significant with increasing holding time suggesting that the grain growth was associated with the grain movements.

### 3.5. Thermal Properties of SPS-Processed Specimens

**Figure 10:**
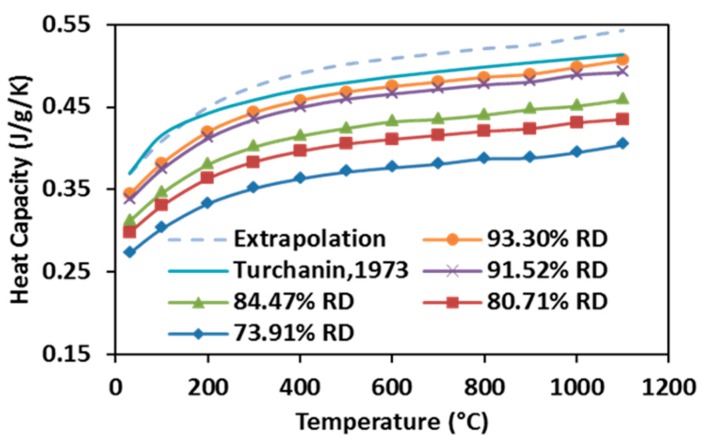
Heat capacities of SPS-processed specimens as a function of temperature.

The heat capacity of specimens SPSed under various processing conditions increase with elevating temperature, as well as with raising the relative density (see Figure 10). Heat capacity first rises rapidly from room temperature to 300 °C, and then it grows slowly until 1100 °C. According to [49], the Debye temperature of stoichiometric ZrC is between 500 and 600 K (200~300 °C), suggesting that the observation from the present study is in accordance with the reported data, as the heat capacity of carbide at low temperatures depends on its Debye temperature. Additionally, for a given volume, a specimen with higher relative density possesses more thermal mass, therefore, more heat is required for a degree of temperature rise. Heat capacities of fully-dense ZrC were extrapolated from the measurements of partially-dense specimens and compared with those calculated by Turchanin et al. using both Debye and Einstein equations [50] in the same graph. It shows that the highest heat capacity obtained from this study is very close to the one reported in the past while the extrapolation is more accurate as the temperature goes over 200 °C.

**Figure 11:**
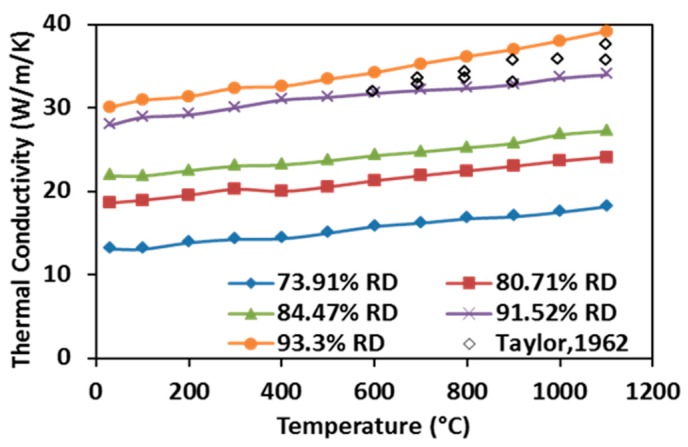
Thermal conductivities of SPS-processed specimens as a function of temperature.

As shown in Figure 11, the thermal conductivities of SPS processed specimens rise with increasing temperature in the tested temperature range. This observation indicates quite unique ZrC properties compared to many other ceramic materials and it has been primarily attributed to the contributions of conduction electron bands and high phonon conductivity in ceramics materials [15]. Additionally, the thermal conductivity is shown to increase with enhancing the relative density because higher relative density is associated with the presence of fewer pores, hence more thermal pathways are present in the processed specimen. Thermal conductivities of the hot-pressed ZrC with very similar relative density (~93.3%) obtained by Taylor were considered to be the highest results that have been reported in the past [30]. These data have been included for comparison in Figure 11 (scatter diamond markers, no data reported for temperature below 600 °C). It appears that the measured thermal conductivities from the SPS-processed specimens are higher than those from the hot-pressed ones. Although the method of characterization between the present study and [30] is very different, the obtained evolutions of thermal conductivities are consistent and the flash method appears to be able to retrieve them at lower temperatures in a shorter time.

SPS-processed specimens exhibited excellent heat capacities and thermal conductivities compared to these reported in the past. The improvements of the thermal properties are most likely due to the reduction of impurities during the SPS process. Impurities are easy to be introduced into powders during manufacturing processes since powders have large surface area and high surface energy. The impurities or secondary atoms usually occupy lattice vacancies or present as interstitials which act as strong scattering centers for phonons and electrons. These impurities are hard to remove during conventional sintering processes. Therefore, both thermal and electrical properties of the sintered product can be negatively influenced. The SPS process provides high electric current enabling the generation of micro-discharges along powder surfaces to remove impurities [51,52] and, in turn, to improve the above-mentioned properties of the final products.

## 4. Conclusions

ZrC pellets with high relative densities have been successfully produced by SPS. Relative densities of obtained specimens were mapped with processing temperature, applied pressure, and holding time to elucidate the effects of these processing parameters on the densification level. Hot pressing and SPS of ZrC were carried out in the conducted comparative study to investigate the different densification mechanisms affecting these two techniques. Higher strain rate sensitivity and lower activation energy are observed for the control SPS compared to those observed for the conventional hot pressing. The causes of these differences have been attributed to the effects of the electric current during SPS processing.

Temperature evolution during SPS of ZrC was implemented by a finite element simulation to characterize the thermal gradient between the die surface and the specimen. The specimen’s actual temperature was verified by correlating the simulated temperatures with respect to the pyrometer measured ones. The specimen’s temperature was then substituted into recently modified models to study the grain growth kinetics in the final stage of SPS, and the grain boundary diffusion was determined to be the major control mechanism. The microscopic examinations of specimen’s cross-sectional area also reflected that the grain growth in the final SPS stage contributes to the closure of the inter-granular pores.

Specific heat capacities and thermal conductivities of the SPS processed specimens were measured from room temperature to 1100 °C using DSC along with LFA. Specimens’ thermal properties were found to increase either with higher relative density or with raising temperature. The thermal properties obtained from the SPS-processed specimens were higher than the reported data retrieved from the hot-pressed samples at the similar relative density level, thereby indicating the impurity cleaning effect during the SPS process.

Both experimental and modeling approaches have been conducted to characterize the hot consolidation of ZrC. The obtained results can be used for future optimization purposes, including the possible design of material structures in a sophisticated way.

## Figures and Tables

**Table 1:** Properties of zirconium carbide used in simulations.

Parameters	Values
Heat capacity, $C_{p}$ (J/kg/K)	$\left(352.8+0.094T-2.55\times 10^{3}T^{-2}\right)\left(1-\theta \right)$
Thermal conductivity, $k_{T}$ (J/m/K)	$\left(\begin{array}{c} 17.82+0.024T-9.39\times \\ 10^{-6}T^{2}+1.68\times 10^{-9}T^{3} \end{array}\right)\left(1-0.5\theta -1.5\theta^{2}\right)$
Electric conductivity, λ (S/m)	$\frac{1}{39.3\times 10^{-8}+76.7\times 10^{-11}T}\left(\frac{1-\theta }{1+2\theta }\right)$

**Table 2:** Optimal creep coefficients.

Parameters	Intermediate Stage			Final Stage		
	*m*	*Q* (*kJ*/*mol*)	*A_0_*	*m*	*Q* (*kJ*/*mol*)	*A*_0_
Hot pressing Control SPS	0.382	653	5.92 × 10^−6^	N/A	N/A	N/A
	0.403	563	6.58 × 10^−6^	0.403	576	6.58 × 10^−6^
